# Psychometric properties of the self-reflection and insight scale (revised) in German-speaking clinical psychology trainees

**DOI:** 10.3389/fpsyg.2026.1733344

**Published:** 2026-10-16

**Authors:** Theresa Hanke, Celina L. Müller, Johanna L. Popp, Martin Weiß, Greta Hanke, Kristin Herrmann

**Affiliations:** 1 Department of Psychiatry and Psychotherapy III, University Hospital Ulm, Ulm University, Ulm, Germany; 2 Department of Psychology I, Würzburg University, Würzburg, Germany; 3 University Hospital Düsseldorf, Heinrich Heine University Düsseldorf, Düsseldorf, Germany

**Keywords:** German adaption, insight, reflective practice, self-assessment, self-reflection, SRIS-R

## Abstract

**Introduction:**

Self-reflection is a core competency for clinical psychologists and mental health professionals. Given its essential role in clinical practice and personal development, it is important to assess self-reflective capacity reliably and efficiently. This study aimed to translate and examine the psychometric properties of the Self-Reflection and Insight Scale-Revised (SRIS-R) for use in German-speaking contexts.

**Methods:**

The SRIS-R was translated using a back-translation procedure. A total of 257 participants completed the questionnaires via an online survey.

**Results:**

Both the original 14-item factor structure and revised German model solutions were tested. Confirmatory factor analyses indicated that the original English factor structure could not be satisfactorily replicated in the German sample. Therefore, exploratory factor analyses using polychoric correlations were conducted and suggested structural differences in the German version. Based on these findings, a parsimonious revised 13-item two-factor scoring solution and a methodologically refined two-factor model including a cross-loading and a residual correlation were examined. The modified 13-item version showed improved internal consistency (α = 0.86 for Self-Reflection and α = 0.84 for Insight). The parsimonious revised 13-item scoring solution retained the conceptual distinction between Self-Reflection and Insight while being feasible for practical questionnaire scoring. Convergent and discriminant validity evidence supported theoretical associations of Self-Reflection with related constructs, particularly self-insight and reflection, whereas Insight showed negative associations with rumination and private self-consciousness. Additional ESEM analyses supported a two-factor representation while indicating relevant item-level cross-loadings. The 13-item structure was subsequently tested without modification in an independent sample (*N* = 161), showing comparable model fit and high factorial similarity across samples. Multigroup analyses further supported scalar invariance across the two samples. Together, these findings provide cross-sample support for the distinction between Self-Reflection and Insight, while indicating remaining structural complexity at the item level. Given the limited support for a general factor, interpretation of the two subscale scores rather than a total score is recommended.

**Discussion:**

However, further research is needed to replicate the revised structure in broader and more diverse German-speaking samples. A culturally adapted and efficient measurement, like the one presented here, is essential for fostering and assessing self-reflective skills in clinical professionals.

## 1 Introduction

Besides working with various methods and interventions, the clinical psychologist as a person is an important tool in therapeutic work. In addition to the development of the therapeutic relationship, case conceptualization, and specific interventions, an essential foundation for competent therapeutic action is the therapist’s ability for self-reflection (EAP - European Association of Psychotherapy, 2013; Rodolfa et al., 2013). Self-reflection is considered a core competency for psychologists (Rice et al., 2022), described as a dynamic and continuing process of development throughout someone’s career as a clinical psychologist (Rodolfa et al., 2013; Rodolfa and Schaffer, 2019; Stevens et al., 2017). This competence is regarded as essential because it enables psychologists to monitor their own thoughts, emotions, and reactions in relation to clinical work, thereby supporting sound professional judgment, ethical decision making, and the quality of the therapeutic relationship (e.g., Bennemann et al., 2021). There has been a major discussion around the definition of self-reflection in general (Nguyen et al., 2014). According to current research, the ability to (self-) reflect consists of several aspects: observation, interpretation, and evaluation of someone’s thoughts, emotions, and behavior (Bennemann et al., 2021; Bennett-Levy, 2006). In practice, this process involves noticing clinically relevant cues, examining their meaning, and assessing their implications for professional action, which in turn provides a basis for ongoing learning and adaptation in clinical practice (Bennemann et al., 2021).

With the implementation of the new law for psychotherapists in Germany, the Approbationsordnung für Psychotherapeutinnen und Psychotherapeuten (Approbationsordnung für Psychotherapeutinnen und Psychotherapeuten (PsychThApproO), 2020), the training of future clinical psychologists has undergone a fundamental restructuring. This legal framework reorganizes the path to licensure by integrating academic and clinical training into a more coherent curriculum, requiring a polyvalent bachelor’s degree in psychology followed by a master’s program in clinical psychology and psychotherapy (Approbationsordnung für Psychotherapeutinnen und Psychotherapeuten (PsychThApproO), 2020). Against this background, the curriculum has shifted from a primarily theory and methodology driven model to a more integrative approach, in which theoretical, practical, and personal development are systematically interlinked. This integrative model serves to support the development of competencies essential for clinical practice, including professional judgment, ethical decision making, and the quality of the therapeutic relationship. A key innovation of the new curriculum is the formal inclusion of self-reflection as a dedicated academic module (Approbationsordnung für Psychotherapeutinnen und Psychotherapeuten (PsychThApproO), 2020). No longer treated as a merely implicit skill, self-reflection is now explicitly taught and systematically cultivated as a core professional competency. Within this module, students are expected to critically examine their own psychotherapeutic actions, assess the strengths and weaknesses of their personality, and consider how these personal characteristics influence the therapeutic relationship and process (Approbationsordnung für Psychotherapeutinnen und Psychotherapeuten (PsychThApproO), 2020; Hanke and Herrmann, 2025). This focus on self-reflection is essential, because it enables trainee psychologists to recognize how their own cognitions, emotions, and reactions may affect clinical decision making and the quality of patient care (Hanke and Herrmann, 2025). Additionally, they are trained to accept constructive feedback and to regulate these internal processes in therapeutic contexts (Hanke and Herrmann, 2025). In this way, self-reflection supports the development of professional competence and a therapeutic relationship that is responsive to patients’ needs rather than to the therapist’s unreflected personal patterns (Sulz, 2015). This curricular reform highlights the centrality of self-reflection as a foundational element of psychotherapeutic competence. By embedding it within formal training, the program aims to foster personal maturity, therapeutic presence, and professional effectiveness among future clinical psychologists (Approbationsordnung für Psychotherapeutinnen und Psychotherapeuten (PsychThApproO), 2020).

To date, there have only been a few studies examining the ability of self-reflection among clinical psychologists. To promote individual’s own reflective competence and advance the professional development of therapists, a reflective attitude is required (Bennemann et al., 2021). This includes interest and curiosity about someone’s own thinking and actions, self-acceptance and tolerance for mistakes, recognizing and tolerating ambiguous, contradictory motives and values (tolerance for ambiguity), acceptance of one’s own affects, and acknowledgment of one’s contribution in the therapeutic relationship (especially in the presence of hostile interpersonal processes; Bennemann et al., 2021). The application and practice of self-reflection ability is an important component in developing and fostering reflective competencies (Bennemann et al., 2021; Bennett-Levy, 2006).

For evaluation how closely achievements align, structured self-assessments can help to compare between a current and a desired performance. Self-assessments as well as empirical research on self-reflection in general are limited (Rice et al., 2022; Ooi et al., 2021). There are two self-assessments available in English, which are recommended for measuring self-reflection skills in healthcare professionals (Ooi et al., 2021). On the one hand, there is the *Reflective Questionnaire* (RQ) from Kember and colleagues (2000) and on the other hand there is the *Self-reflection and Insight Scale* (SRIS; Grant et al., 2002, revised form: Banner et al., 2023). The SRIS (Grant et al., 2002) was developed to examine levels of self-reflection, promoted as a measurement assessing private self-consciousness. The original version is a 20-item questionnaire, comprising two subscales (*Self-Reflection* (12 items), *Insight* (8 items)) based on a sample of 260 undergraduate psychology students. The answers are rated on a 6-point Likert scale (1 = strongly disagree, 6 = strongly agree), whereas higher scores are associated with a stronger self-reflection ability. Good to excellent internal consistency (α = 0.91 for *Self-Reflection*; α = 0.87 for *Insight*), and a good convergent and divergent validity has been reported (Grant et al., 2002). The SRIS has been validated on a homogeneous sample and has been adapted for use in various healthcare professions (Pai, 2015, 2016; Lowe et al., 2007; Roberts and Stark, 2008). Whereas *Self-Reflection* is defined as “the inspection and evaluation of one’s thoughts, feelings and behavior”, *Insight* is described as “the clarity of understanding the one’s thoughts, feelings and behavior” (Grant et al., 2002). In 2023, Banner and colleagues published a revised version of the SRIS (SRIS-R). Whereas the original SRIS consists of 20 items, with 12 items assessing Self-Reflection and 8 items assessing Insight, the SRIS-R is a shortened 14-item version with two equally sized subscales (7 items each). In their validation study, Banner et al. (2023) confirmed the reliability of the original scale and showed that the revised form retained satisfactory psychometric properties. The SRIS-R was selected for the present study because it provides a more economical assessment, reduces respondent burden, and preserves the two core dimensions of self-reflection and insight.

The 20-item solution (Grant et al., 2002) demonstrated good internal consistency (*α* = 0.89) as a total score, whereas the revised short version (Banner et al., 2023) showed good internal consistency (*α* = 0.88) for total, and good, respective acceptable internal consistency across both subscales (*Self-Reflection*: *α* = 0.91; *Insight α* = 0.79). Convergent validity was assessed using the self-reflection subscale of the Competences of Professional Psychology Rating Scale (COPPR Scales, Rice et al., 2022), while divergent validity was evaluated using the perceived knowledge subscale of the CFI-9 (McIlveen et al., 2013). The SRIS-R demonstrated a good divergent (*r* = 0.06–0.09) and convergent (*r* = 0.27–0.35) validity. Furthermore, across both, the total score as well as the subscales scores, no significant correlation with years of registration as a psychologist was shown (*r* = 0.05–0.12).

So far, as described in detail above, there is little research on the factors influencing self-reflection ability, and there is currently no German-language questionnaire that assesses self-reflection ability among clinical psychologists. Given the changing study program in clinical psychology, which accompanies with predefined content in teaching self-reflection skills, there is an urgent need for a valid measure of self-reflection for clinical psychologists. Grant et al. (2002) conceptualized Self-Reflection and Insight as related but distinct dimensions of self-focused cognition, although their empirical separation is not always clear-cut. For translated and culturally adapted versions, linguistic nuances and differences in item functioning may affect how items relate to the underlying subscales. Therefore, the present study evaluated the German SRIS-R as an adapted measure and empirically examined whether the original item structure could be retained in the German sample.

Accordingly, this study aimed to examine the psychometric properties and factorial validity of the first German-language adaptation of the *Self-Reflection and Insight Scale - Revised* (SRIS-R) in a sample of German-speaking psychology students. We hypothesized that the German SRIS-R would show the same two-factor structure as the original, with items loading on the intended Self-Reflection and Insight factors (Grant et al., 2002; Banner et al., 2023). For convergent validity, the *Rumination–Reflection Questionnaire* (RRQ; Trapnell and Campbell, 1999; German version: Schütz, 2000), and the *Self-Consciousness-Scale* (SCS; Fenigstein et al., 1975; German version: Heinemann, 1979) were selected based on their conceptual overlap with self-reflection, private self-consciousness, and related self-focused thought, and the *Selbstkenntnismotivationsskala* (SIM; Heine et al., 2024) was added as an additional measure of self-knowledge motivation. Accordingly, we expected positive associations with these measures, whereas only weak associations were anticipated with the *Career Future Inventory* (CFI; Rottinghaus et al., 2012; German version: Spurk and Volmer, 2013) perceived knowledge of the job market subscale (Banner et al., 2023).

Regarding associations with external constructs, Self-Reflection was expected to show positive associations with conceptually related tendencies toward reflection, self-focused attention, and motivation for self-knowledge. Insight was expected to show a distinct pattern, particularly negative associations with maladaptive forms of self-focused attention such as rumination and private self-consciousness. Associations with cognitive flexibility were expected to be comparatively weaker, given its greater conceptual distance from the core processes assessed by the SRIS-R. As no specific effect-size thresholds had been specified *a priori*, the magnitude of these associations was evaluated descriptively rather than against predefined criteria.

The present study aimed to examine the psychometric properties of the German adaptation of the SRIS-R and to provide initial evidence regarding the interpretation of SRIS-R scores in a German-speaking clinical psychology student sample. Specifically, confirmatory and exploratory factor analyses were conducted to examine the factorial structure of the German SRIS-R, and associations with theoretically related and unrelated constructs were investigated to evaluate the conceptual distinctiveness of Self-Reflection and Insight.

## 2 Materials and methods

### 2.1 Participants

Sample-size requirements for factor analysis depend on multiple characteristics, including the number of indicators and factors, magnitude of factor loadings and communalities, factor correlations, and distributional properties of the indicators. Accordingly, no universal participant-to-item ratio was assumed to determine adequacy. Given the relatively short 14-item instrument, the expected two-factor structure, and the use of ordinal-appropriate estimation, a target sample of approximately *N* = 250 was considered adequate for the initial psychometric evaluation (Mundfrom et al., 2005; Wolf et al., 2013). The stability of the resulting structure was subsequently examined in an independent sample (*N* = 161). This aligns with previous validation studies (Banner et al., 2023; Grant et al., 2002). Additionally, a power analysis for medium-sized correlations (*r* = 0.30, *α* = 0.05, power = 0.80) indicates that a minimum of 85 participants would suffice for analyses examining associations with external measures, confirming that the available sample provides adequate statistical power.

Inclusion criteria comprised current enrollment in a Bachelor’s or Master’s program in psychology and clinical psychology at a German university. Participants were recruited voluntarily between April 2025 and July 2025. Recruitment took place after seminars or in return for research participation credit, which formed part of the study requirements.

Of the 280 participants that started the survey, a total of *n* = 257 participants completed the survey, while 23 participants dropped out during data collection.

Of these participants, 249 were pursuing a Bachelor’s degree and 8 a Master’s degree (6 Master’s program in clinical psychology; 2 general Master’s program) of psychology. The sample was predominantly female (84%). The participants’ mean age was 21.93 years (SD = 4.11).

### 2.2 Procedure

The German version of the SRIS-R was developed using a multistep forward-backward translation procedure (see Figure 1). First, the original English items were independently translated into German by two native English speakers with professional backgrounds in psychology and neuroscience. The two forward translations were subsequently compared and consolidated into a preliminary German version. Discrepancies in wording or meaning were discussed jointly by the translators and the authors until consensus on the final wording was reached. The resulting German version was then independently back-translated into English by two German native speakers with professional backgrounds in psychology and medicine. The back-translations were compared with the original English version, and any discrepancies were again discussed among the translators and authors and resolved by consensus. Finally, the resulting German items were reviewed by 10 independent experts, all psychologists who had not been involved in the translation process, who evaluated the items with regard to comprehensibility and wording. Their feedback was considered in finalizing the German version used in the present study.

**Figure 1:**
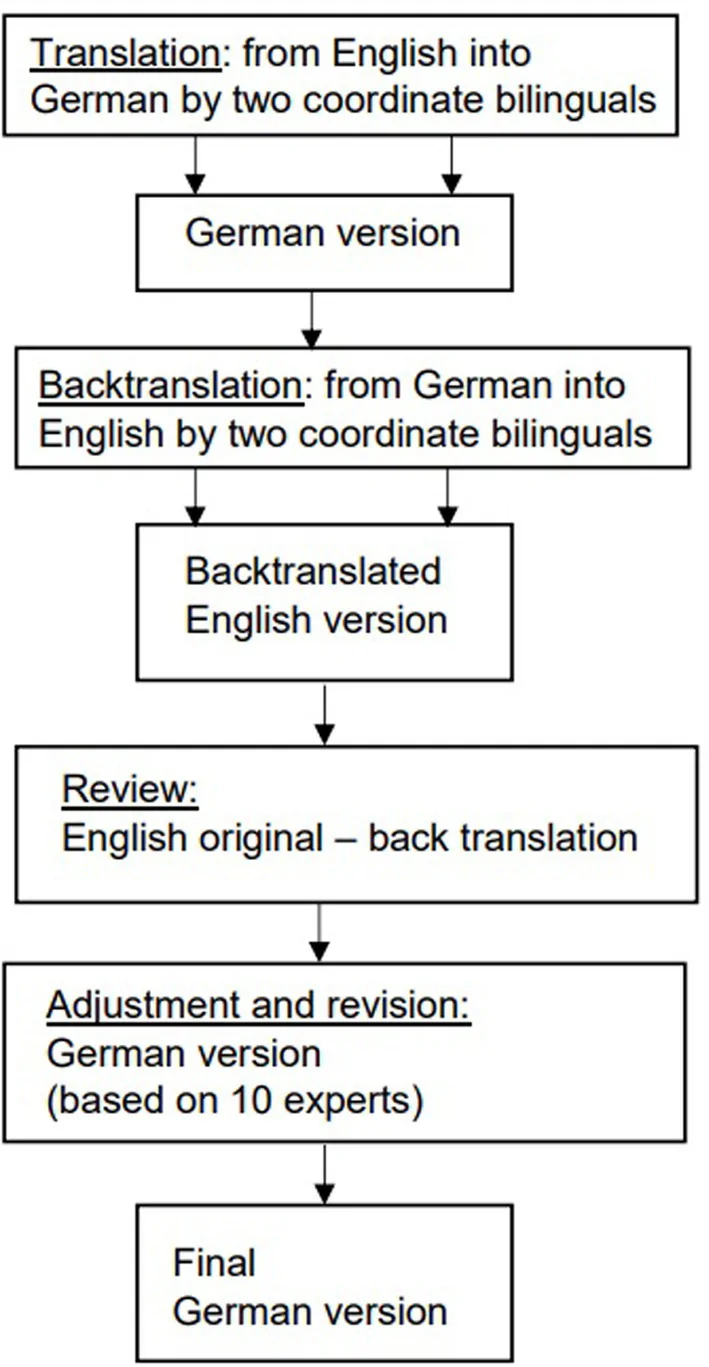
Translation procedure of the German SRIS-R of SRIS-R.

Subsequently, the study was advertised at German universities that offer psychology programs. Participants provided informed consent and then completed demographic questions as well as study measures (containing the German version of SRIS-R and questionnaires for convergent and divergent validity) via an online survey (SoSci Survey), of an approximately 30 min duration. The study was approved by the ethics committee of the department of psychology at the University of Würzburg (approval number GZ 2024–70, on 03.02.2025). The study and its general analysis plan were registered on the Open Science Framework.^1^

### 2.3 Measures

*SRIS-R.* The 14-items version of the SRIS-R (Banner et al., 2023) consists of two subscales -*Self-Reflection* and *Insight*, each comprising 7 items. As previously described, the SRIS-R demonstrated acceptable to good internal consistency, as well as good convergent and divergent validity.

*CFI*. As a measure of divergent validity, participants completed the CFI (Rottinghaus et al., 2012; German version: Spurk and Volmer, 2013). The instrument captures three theoretically distinct yet related dimensions of career-related cognitions: *Career Adaptability*, *Career Optimism*, and *Perceived Knowledge of the Job Market.* The scale comprises 25 items: 11 each for *Career Adaptability* and *Career Optimism*, and 3 for *Perceived Knowledge* of the Job Market. Items are rated on a six-point Likert scale ranging from 1 (“strongly disagree”) to 6 (“strongly agree”). The measure demonstrated satisfactory test–retest reliability over a three-week interval, with internal consistency estimates ranging from *α* = 0.73 to 0.87 across subscales. Evidence for construct validity was also reported, with positive correlations between *Career Adaptability* and the Big Five trait *Conscientiousness* (supporting convergent validity) and low correlations with unrelated constructs such as the *Enterprising* and *Artistic* subscales of the Strong Interest Inventory (SII), supporting discriminant validity (Rottinghaus et al., 2005). A German adaptation of the CFI was developed and validated by Spurk and Volmer (2013) in samples of mid-career employees working in small and medium-sized enterprises. Their results confirmed the three-factor structure and demonstrated internal consistencies (*α* = 0.78–0.90) comparable to the original version (Spurk and Volmer, 2013). Convergent validity was indicated by positive correlations of all three subscales with career success, while discriminant validity was supported by negative associations with Neuroticism (Spurk and Volmer, 2013). As optimism and adaptability were expected to be related to self-reflection, divergent validation in this study was, in line with the original study (Banner et al., 2023), limited to the “perceived knowledge” subscale, which include, for example, items assessing the understanding of employment trends.

*RRQ*. The Rumination–Reflection Questionnaire (RRQ; Trapnell and Campbell, 1999), was included as a convergent validity measure and assesses two distinct dimensions of private self-focused thought: *Rumination* and *Reflection*. Each subscale consists of 12 items rated on a five-point Likert scale ranging from 1 (“strongly disagree”) to 5 (“strongly agree”). Exploratory and confirmatory analyses by Trapnell and Campbell (1999) supported a two-factor structure, with only a small correlation between the subscales (*r* = 0.22), indicating distinct underlying constructs. Internal consistency was high for both *Reflection* (*α* = 0.91) and *Rumination* (*α* = 0.90). Evidence for construct validity included positive correlations with private self-consciousness (*r* = 0.59 for *Reflection*; *r* = 0.43 for *Rumination*). Further, *Rumination* showed convergent associations with *Neuroticism* (*r* = 0.57–0.64) and divergent relations with *Extraversion* (*r* = −0.15), while *Reflection* correlated positively with *Openness to Experience* (*r* = 0.61–0.68) and only weakly with *Neuroticism* (*r* = 0.15), confirming the theoretical distinctness of the subscales (Trapnell and Campbell, 1999).

For the present study, the German adaptation of the RRQ by Schütz (2000) was used. Participants rated all 24 items on a five-point scale from 1 (“strongly disagree”) to 5 (“strongly agree”).

*SCS*. The SCS (Fenigstein et al., 1975; German version: Heinemann, 1979) was used as a convergent measurement. The SCS was developed to assess individual differences in self-focused attention and related affective responses. It comprises three subscales: *Private Self-Consciousness*, *Public Self-Consciousness*, and *Social Anxiety*. The scale consists of 23 items rated on a five-point Likert scale from 1 (“strongly disagree”) to 5 (“strongly agree”).

Factor analytic results supported a three-factor structure, with all items loading at least 0.40 on their intended factor (Fenigstein et al., 1975). Intercorrelations among the subscales indicated that *Public Self-Consciousness* correlated moderately with *Private Self-Consciousness* (*r* = 0.23–0.26) and *Social Anxiety* (*r* = 0.20–0.21), while *Private Self-Consciousness* and *Social Anxiety* were uncorrelated, supporting their discriminant validity. Test–retest reliability over a two-week interval was satisfactory, with internal consistency estimates of *α* = 0.84 (*Public Self-Consciousness*), *α* = 0.79 (*Private Self-Consciousness*), and *α* = 0.73 (*Social Anxiety*) (Fenigstein et al., 1975).

A German adaptation of the SCS was developed and validated by Heinemann (1979). Factor analyses confirmed the original three-factor structure and similar intercorrelation patterns among subscales (*Public Self-Consciousness - Private Self-Consciousness*: *r* = 0.39; *Public Self-Consciousness - Social Anxiety*: *r* = 0.29; *Private Self-Consciousness - Social Anxiety*: n.s.). Test–retest reliability over a four-week interval demonstrated good internal consistencies (*α* = 0.83, 0.77, and 0.82 for *Public Self-Consciousness*, *Private Self-Consciousness*, and *Social Anxiety*, respectively) (Heinemann, 1979).

The subscale private consciousness was utilized for convergent validation in this study.

*SIM. The Selbstkenntnismotivationsskala* (SIM; Heine et al., 2024) was originally developed in German to assess an individual’s motivation to gain true and accurate self-knowledge and was included as a convergent validity. Confirmatory factor analyses of this 5-item scale supported a unidimensional structure, which was replicated after both 8 weeks and 1 year. The SIM demonstrated high internal consistency (*α* = 0.81 at baseline, *α* = 0.85 after 2 months, and *α* = 0.85 after 12 months) and strong test–retest reliability (*r* = 0.77 between T1 and T2). In a larger, nationally representative sample of the German population (*N* = 1,612), the scale again showed excellent internal consistency (*α* = 0.88). Evidence for construct validity was provided through positive correlations with related personality constructs such as Openness to Experience, Curiosity, and Private Self-Consciousness, supporting convergent validity (Heine et al., 2024).

In the present study, participants completed the 5-item SIM using a six-point Likert scale ranging from 1 (“strongly disagree”) to 6 (“strongly agree”). The SIM was employed to examine convergent validity.

### 2.4 Statistical analysis

All statistical analyses were conducted in R version 4.4.2 (R Core Team, 2024) using RStudio (Version 2025.09.2) as the integrated development environment. Confirmatory factor analyses, bifactor models, and exploratory structural equation models (ESEM) were estimated using lavaan (Version 0.6–19; Rosseel, 2012). Exploratory factor analyses and reliability analyses were conducted using psych (Version 2.5.6) and GPArotation (Version 2025.3.1), with nFactors (Version 2.4.1.2) additionally used for factor-retention analyses. SEM-based psychometric indices were calculated using semTools (Version 0.5–7), and bifactor-specific indices were obtained using BifactorIndicesCalculator (Version 0.2.2). Data preparation and management were performed using tidyverse (Version 2.0.0). The psychometric properties of the German SRIS-R were examined in several sequential steps. First, descriptive item analyses and internal consistency estimates were computed for the 14-item German version. Second, the original factorial assumptions were evaluated using confirmatory factor analysis (CFA) by testing a one-factor model, the original two-factor model proposed by Banner et al. (2023), and a bifactor model. Because all SRIS-R items were assessed using ordinal Likert-type response formats, confirmatory factor analyses were estimated using the weighted least squares mean and variance adjusted estimator (WLSMV) with theta parameterization. Model fit was evaluated using the comparative fit index (CFI), Tucker-Lewis index (TLI), root mean square error of approximation (RMSEA) including 90% confidence intervals, and the standardized root mean square residual (SRMR). Following common recommendations by Hu and Bentler (1999) and Rutkowski and Svetina (2014), CFI and TLI values ≥ 0.90 were interpreted as acceptable and ≥ 0.95 as good, RMSEA values ≤ 0.08 as acceptable and ≤ 0.06 as good, and SRMR values ≤ 0.08 as acceptable. Because the original structure demonstrated insufficient fit, an exploratory phase followed. Prior to exploratory factor analyses (EFA), the suitability of the data for factor analysis was evaluated using the Kaiser-Meyer-Olkin (KMO) criterion and Bartlett’s test of sphericity. Because the SRIS-R items consisted of ordinal Likert-type variables and several items showed non-normal distributions, EFAs were conducted using polychoric correlation matrices. Factors were extracted using minimum residual estimation with oblimin rotation, allowing correlations between factors. The number of factors was determined based on parallel analysis using randomly resampled data, empirical model fit indices, and theoretical interpretability. Factor loadings ≥ 0.32 were considered salient following recommendations by Tabachnick and Fidell (2007). Cross-loadings were defined as secondary loadings ≥ 0.30.

Based on the exploratory findings, several revised CFA models were subsequently estimated, including a parsimonious revised two-factor model, a revised two-factor model with a theoretically justified cross-loading, and a methodologically refined two-factor model additionally allowing a residual correlation between two semantically overlapping items.

To address the dependence between exploratory and confirmatory analyses conducted within the same sample, two additional analytical steps were implemented. First, the factor structure was examined using Exploratory Structural Equation Modeling (ESEM; Asparouhov and Muthén, 2009; Marsh et al., 2014). ESEM combines the freely estimated loadings characteristic of EFA with the model-testing and fit-index framework of CFA. Models were estimated using WLSMV based on polychoric correlations and geomin-oblique rotation. Second, to cross-validate the findings, the final 13-item model was tested without modification in an independent sample of an ongoing study (*N* = 161). This sample comprised bachelor’s and master’s students in clinical psychology as well as psychotherapists in training, recruited at the University of Würzburg from cohorts distinct from those included in the primary study.

Internal consistency was evaluated using Cronbach’s alpha, standardized alpha, McDonald’s omega, and model-based composite reliability coefficients derived from CFA models. Item-total correlations and alpha-if-item-deleted statistics were additionally examined. Convergent and discriminant validity evidence was explored through correlations with external validation measures, including the RRQ, SCS, SIM, and the perceived knowledge subscale of the CFI. Because the data consisted of ordinal Likert-type variables and several variables showed non-normal distributions, Spearman rank-order correlations were calculated. To compare the psychometric behavior of the original (proposed by Banner et al., 2023) and revised German structures, correlations were computed for both scoring approaches.

## 3 Results

The back-translation procedure led to only minor wording adjustments, the final German version is provided in the Electronic Supplementary Material.

### 3.1 Results of the original structure

#### 3.1.1 Descriptive statistics of the original structure

Descriptive item statistics for the original 14-item German SRIS-R are presented in Supplementary Table S1. Item means ranged from *M* = 3.67 (SD = 1.16) to *M* = 5.34 (SD = 0.83), indicating moderate to high endorsement frequencies across items. Several items, particularly Self-Reflection items, showed pronounced negative skewness, suggesting ceiling tendencies.

#### 3.1.2 Internal consistency of the original structure

Internal consistency estimates for the original SRIS-R structure were acceptable overall (see Table 1). Omega total (*ω_t_* estimates were higher across scales).

**Table 1:** Internal consistency for the original SRIS-R structure.

SRIS-R	Cronbach’s *α*	Standardized *α*	*ω* total
Total score	0.764	0.794	0.869
Self-reflection	0.703	0.790	0.908
Insight	0.808	0.799	0.892

*α*, Cronbach’s alpha; standardized; *α*, standardized Cronbach’s alpha; ω total, McDonald’s omega total. Higher values indicate greater internal consistency.

#### 3.1.3 Confirmatory factor analyses of the original structure

All model fit indices for the initially tested CFA models were shown in Table 2. A one-factor CFA demonstrated very poor fit to the data. The original two-factor structure proposed by Banner et al. (2023) also showed poor fit, indicating that the original English factor structure could not be directly replicated in the German sample. Although the bifactor model demonstrated comparatively favorable global fit indices, bifactor-specific diagnostics did not support a substantively meaningful dominant general factor. The explained common variance attributable to the general factor was low and omega hierarchical for the general factor was modest, indicating that only a limited proportion of reliable variance could be attributed to a common overarching construct. In contrast, the group factors, particularly Insight, retained substantial reliable variance beyond the general factor with Insight.

**Table 2:** Model fit indices for the initially tested CFA models.

Model	*χ* (df)	CFI	TLI	RMSEA [90% CI]	SRMR	ECV	ω_h_ general factor	ω_h_ Subscales	H	FD
One-factor model	1731.32 (77)	0.377	0.264	0.225 [0.214, 0.237]	0.211					
Original two-factor model	805.68 (76)	0.725	0.671	0.140 [0.128, 0.153]	0.144					
Bifactor model	154.35 (63)	0.966	0.950	0.078 [0.062, 0.094]	0.060	0.38	0.29	IN = 0.679 SR = 0.359	IN = 0.883 SR = 0.776	IN = 0.941 SR = 0.875

CFI, Comparative Fit Index; TLI, Tucker-Lewis Index; RMSEA, Root Mean Square Error of Approximation; SRMR, Standardized Root Mean Square Residual; ECV, Explained Common Variance; ωh, omega hierarchical; H, construct replicability coefficient; FD, factor determinacy. IN, Insight; SR, Self-Reflection. RMSEA values are reported with 90% confidence intervals. Higher values of CFI and TLI and lower values of RMSEA and SRMR indicate better model fit.

### 3.2 Exploratory factor analyses

Because the original factorial structure showed insufficient fit, exploratory factor analyses were conducted using polychoric correlations. The data were suitable for factor analysis, with an overall KMO value of 0.83 and a significant Bartlett’s test of sphericity, *χ*^2^ (91) = 1662.04, *p* < 0.001. Parallel analysis based on polychoric correlations suggested a three-factor solution. Therefore, both a three-factor and a theoretically more parsimonious two-factor solution were inspected. The three-factor solution showed better exploratory fit, *χ*^2^ (52) = 134.27, CFI = 0.948, TLI = 0.908, RMSEA = 0.078, 90% CI [0.062, 0.095], BIC = −154.28, and accounted for 54.35% of the variance. The two-factor solution showed lower fit, *χ*^2^ (64) = 261.42, CFI = 0.874, TLI = 0.820, RMSEA = 0.109, 90% CI [0.096, 0.124], BIC = −93.73, and accounted for 48.66% of the variance.

However, the three-factor solution split self-reflection-related content into two highly related subcomponents (*r* = 0.52; see also Supplementary Table S2 for factor loadings), whereas the two-factor solution was more consistent with the theoretical SRIS-R distinction between Self-Reflection and Insight. In the two-factor EFA, Item 1 showed no salient loading on either factor, whereas Item 5 showed a cross-loading pattern (see Table 3). Based on the EFA results and theoretical interpretability, a revised two-factor structure was developed and subsequently tested using CFA.

**Table 3:** Exploratory factor analysis loadings for the two-factor solution based on polychoric correlations.

Item	Factor 1 (Self-Reflection)	Factor 2 (Insight)	Primary loading	Secondary loading	Factor suggestion
Item 1	0.082	0.224	0.224	0.082	No salient loading
Item 2	0.558	0.255	0.558	0.255	Factor 1
Item 3	−0.122	0.756	0.756	0.122	Factor 2
Item 4	0.708	−0.054	0.708	0.054	Factor 1
Item 5	0.472	0.375	0.472	0.375	Cross-loading
Item 6	0.838	0.012	0.838	0.012	Factor 1
Item 7	−0.060	0.684	0.684	0.060	Factor 2
Item 8	0.021	0.658	0.658	0.021	Factor 2
Item 9	0.730	−0.052	0.730	0.052	Factor 1
Item 10	0.063	0.732	0.732	0.063	Factor 2
Item 11	0.717	−0.089	0.717	0.089	Factor 1
Item 12	0.669	0.080	0.669	0.080	Factor 1
Item 13	0.018	0.868	0.868	0.018	Factor 2
Item 14	0.701	−0.109	0.701	0.109	Factor 1

Loadings are pattern coefficients from minimum residual factor analysis with oblimin rotation. A loading threshold of 0.32 was used for interpretation; cross-loading was defined as a secondary loading ≥ 0.30.

### 3.3 Revised confirmatory factor models

Model fit indices for all revised and exploratory CFA models are presented in Table 4. The exploratory three-factor model demonstrated better fit than the original two-factor structure. However, because the three-factor solution split self-reflection-related content into two correlated components and was less consistent with the theoretical distinction between Self-Reflection and Insight, subsequent model refinement focused on a revised two-factor solution.

**Table 4:** Model fit indices for revised and exploratory CFA models.

Model	*χ*^2^ (df)	CFI	TLI	RMSEA [90% CI]	SRMR
Exploratory three-factor model	339.19 (62)	0.893	0.865	0.096 [0.081, 0.111]	0.087
Parsimonious Revised Two-factor Model	430.58 (64)	0.858	0.827	0.120 [0.107, 0.134]	0.103
Revised two-factor model with cross-loading	283.61 (63)	0.915	0.894	0.109 [0.094, 0.124]	0.082
Methodologically Refined Two-Factor Model	245.35 (62)	0.929	0.911	0.092 [0.077, 0.107]	0.077

All models were estimated using WLSMV estimation for ordinal indicators. The Parsimonious Revised Two-factor Model represents the practically applicable 13-item scoring solution, excluding Item 1 and assigning Item 5 exclusively to the Self-Reflection factor. The revised two-factor model with cross-loading additionally allowed Item 5 to load on both Self-Reflection and Insight. The Methodologically Refined Two-Factor Model further included a residual correlation between Items 11 and 14 to account for local item dependency due to semantic overlap. The exploratory three-factor model was derived from exploratory factor analyses and separated self-reflection-related content into two correlated components.

In the initial 13-item revised two-factor model (*Parsimonious Revised Two-factor Model*), Item 1 was removed because it did not show a salient loading on either factor in the exploratory analyses. Item 5 was assigned to the Self-Reflection factor because it demonstrated a stronger primary loading on this factor and conceptually reflects behavioral aspects of self-reflection. The resulting revised 13-item structure therefore included Items 2, 4, 5, 6, 9, 11, 12, and 14 for the Self-Reflection subscale and Items 3, 7, 8, 10, and 13 for the Insight subscale. The revised two-factor model improved fit relative to the original structure but still remained below recommended thresholds.

Inspection of modification indices and theoretical item content further suggested that Item 5 captured aspects of both behavioral self-reflection and insight. Adding a theoretically justified cross-loading of Item 5 on the Insight factor substantially improved model fit.

The methodologically refined two-factor model additionally included a residual correlation between Items 11 and 14 (MI = 53.04, EPC = −0.61), the standardized residual correlation between Items 11 and 14 was 0.48 [SE = 0.05, 95% CI (0.37, 0.58), *p* < 0.001], reflecting local item dependency likely due to similar wording and semantic overlap. This final exploratory model demonstrated the best empirical fit among the tested two-factor solutions while retaining theoretical interpretability.

Although the *Methodologically Refined Two-Factor Model* showed superior fit, models including cross-loadings and correlated residuals are difficult to implement in routine questionnaire scoring. The *Methodologically Refined Two-Factor Model* was primarily used to characterize localized areas of model misspecification and structural complexity. Therefore, the *Parsimonious Revised Two-factor Model* was retained as the recommended practical scoring solution. Standardized factor loadings of the *Parsimonious Revised Two-factor Model* and the *Methodologically Refined Two-Factor Model* are presented in the Supplementary Table S3. In the *Methodologically Refined Two-Factor Model* all primary factor loadings were statistically significant (*p*s < 0.001) and ranged from 0.499 to 0.878. Item 5 showed a secondary loading on Insight (*λ* = 0.359), supporting the assumption that the item captures both behavioral self-reflection and self-understanding. The latent correlation between Self-Reflection and Insight was small and non-significant (*r* = 0.052, *p* = 0.315), indicating substantial discriminant separation between both constructs. In the *Parsimonious Revised Two-factor Model*, all standardized factor loadings were statistically significant (*p*s < 0.001) and ranged from 0.549 to 0.876. The latent correlation between Self-Reflection and Insight was small but statistically significant (*r* = 0.122, *p* = 0.013), indicating substantial but not complete separation between both constructs.

However, because models including cross-loadings and correlated residuals are difficult to implement in routine questionnaire scoring, the Parsimonious Revised Two-factor Model was selected for subsequent analyses of internal consistency and relationships with external measures.

### 3.4 ESEM and independent cross-validation

The one-factor ESEM model showed poor fit (CFI = 0.377, TLI = 0.264, RMSEA = 0.225, SRMR = 0.211). The two-factor ESEM solution fit the data substantially better, *χ*^2^ (64) = 248.59, CFI = 0.930, TLI = 0.901, RMSEA = 0.111, 90% CI [0.096, 0.126], SRMR = 0.057, and thus achieved virtually the same level of fit as the methodologically refined CFA model without requiring any post-hoc modifications. These findings suggest that the inadequate fit of the original CFA structure may partly reflect the restrictive assumption of zero cross-loadings rather than incorrect item allocation alone. In the two-factor ESEM solution, Item 1 showed no substantial loading on either factor (0.22/0.09), whereas Item 5 loaded substantially on both factors (0.40/0.47), empirically supporting the theoretically proposed cross-loading. The factors were virtually uncorrelated (*r* = 0.02). To cross-validate the findings, the final 13-item model was tested without modification in an independent sample (*N* = 161). Model fit was comparable to that observed in the derivation sample, *χ*^2^ (62) = 170.24, CFI = 0.927, TLI = 0.908, RMSEA = 0.118, 90% CI [0.098, 0.139], SRMR = 0.090 (derivation sample: CFI = 0.929, TLI = 0.911, SRMR = 0.077). The two-factor ESEM solutions across the two samples showed high factorial similarity (Tucker’s φ = 0.95 and 0.98, respectively), and a multigroup model supported scalar measurement invariance (ΔCFI = 0.001, Δ*χ*^2^ (36) = 46.47, *p* = 0.114). Thus, the model decisions derived from the original sample do not appear to be attributable to sample-specific characteristics.

### 3.5 Internal consistency of the parsimonious revised two-factor model

The parsimonious revised 13-item two-factor scoring structure demonstrated good internal consistency for both subscales. Compared with the original structure (14-item), internal consistency improved for both Self-Reflection and Insight. For the original 14-item structure, Cronbach’s alpha was 0.703 for Self-Reflection and 0.808 for Insight. For the revised 13-item structure, Cronbach’s alpha increased to 0.858 for Self-Reflection and 0.837 for Insight. McDonald’s omega total coefficients were *ω* = 0.912 for Self-Reflection and *ω* = 0.880 for Insight. Model-based composite reliability estimates derived from the revised two-factor CFA were CR = 0.866 for Self-Reflection and CR = 0.844 for Insight. Average variance extracted values were acceptable for both constructs, with *AVE* = 0.504 for Self-Reflection and *AVE* = 0.601 for Insight. All internal consistency estimates are presented in Table 5.

**Table 5:** Internal consistency estimates for the original and parsimonious revised SRIS-R structures.

Model	Scale	*α*	Standardized *α*	*ω* Total	CR	AVE
Original structure (14-item)	Total score	0.764	0.794	0.869	—	—
	Self-reflection	0.703	0.790	0.908	0.767	0.522
	Insight	0.808	0.799	0.892	0.839	0.496
Revised structure (13-item)	Total score	0.796	0.806	0.879	—	—
	Self-reflection	0.858	0.862	0.912	0.866	0.504
	Insight	0.837	0.837	0.880	0.844	0.601

CR, Composite Reliability; AVE Average Variance Extracted. CR and AVE are reported for the latent subscales. AVE was interpreted for the original and parsimonious revised two-factor models because both follow a simple factor structure.

#### 3.5.1 Item analyses of the parsimonious revised two-factor model

All item statistics for the *Parsimonious Revised Two-Factor Model* are presented in Table 6. Corrected item-total correlations in the parsimonious revised model ranged from *λ* = 0.482 to *λ* = 0.740 for Self-Reflection and from *λ* = 0.541 to *λ* = 0.741 for Insight. Removing individual items did not meaningfully improve internal consistency. Item 5 showed the lowest corrected item-total correlation within Self-Reflection, *r* = 0.482; however, deleting the item only marginally increased alpha. Given its conceptual relevance for behavioral self-reflection, the item was retained in the Self-Reflection subscale.

**Table 6:** Item statistics for the revised self-reflection and insight subscales.

Scale	Item	Corrected item-total correlation	*α*, if item deleted
Self- reflection	Item 2	0.579	0.843
	Item 4	0.626	0.838
	Item 5	0.482	0.854
	Item 6	0.740	0.825
	Item 9	0.632	0.838
	Item 11	0.621	0.839
	Item 12	0.575	0.847
	Item 14	0.609	0.840
Insight	Item 3	0.653	0.801
	Item 7	0.605	0.814
	Item 8	0.541	0.830
	Item 10	0.660	0.799
	Item 13	0.741	0.775

#### 3.5.2 Discriminant validity

HTMT analyses indicated good discriminant validity between Self-Reflection and Insight. The HTMT value was lower for the parsimonious revised structure than for the original structure, with HTMT = 0.13 for the revised model compared with HTMT = 0.23 for the original model.

#### 3.5.3 External validity

Spearman correlations with external measures are presented in Table 7. The revised Self-Reflection subscale showed moderate positive associations with the Self-Insight Measure (SIM; *ρ* = 0.491) and RRQ Reflection (*ρ* = 0.392), as well as smaller positive associations with private self-consciousness (*ρ* = 0.252) and RRQ Rumination (*ρ* = 0.223). The revised Insight subscale showed negative associations with RRQ Rumination (*ρ* = −0.306) and private self-consciousness (*ρ* = −0.229), whereas associations with SIM, RRQ Reflection, and CFI Knowledge were close to zero.

**Table 7:** Spearman correlations of the original and parsimonious revised SRIS-R scoring structures with external validation measures.

Model	Factor	SIM	SCS private	RRQ reflection	RRQ rumination	CFI knowledge
Original 14-item scoring	Self-Reflection	0.443**	0.280**	0.279**	0.183**	−0.043
	Insight	0.112	−0.176**	0.022	−0.257**	0.014
Revised 13-item scoring	Self-Reflection	0.491**	0.252**	0.392**	0.223**	0.042
	Insight	0.039	−0.229**	−0.037	−0.306**	−0.023

SIM, Self-Insight-Motivation Measure; SCS Private, Self-Consciousness Scale Subscale Private; RRQ, Rumination-Reflection Questionnaire; CFI Knowledge, Career Future Inventory, Knowledge subscale. Spearman correlations are reported. **p < 0.01.

Compared with the original scoring structure, the revised scoring structure showed a more differentiated nomological pattern, particularly stronger associations of Self-Reflection with SIM and RRQ Reflection and stronger negative associations of Insight with RRQ Rumination and private self-consciousness.

## 4 Discussion

Therapists in Germany now receive explicit training in self-reflection, which has been recognized as a key professional competency rather than merely an implicit skill. However, no validated tool is available for (clinical) psychologists to assess their own self-reflection. The present study contributes to closing this gap by evaluating the psychometric properties of a German version of a self-reflection instrument within a psychology student sample. Confirmatory factor analyses indicated that the original English factor structure proposed by Banner et al. (2023) could not be satisfactorily replicated in the German sample. In particular, the originally proposed two-factor solution showed poor model fit and several indications of structural misspecification. Consequently, exploratory factor analyses were conducted to identify a factorial structure more suitable for the German adaptation. These analyses suggested both a theoretically interpretable revised two-factor solution and a methodologically refined two-factor solution including a cross-loading of Item 5 and a residual correlation between Items 11 and 14. Overall, the findings supported a differentiated conceptualization of Self-Reflection and Insight while also indicating that some items may function differently in the German version compared to the original English instrument.

### 4.1 Psychometric refinement and structural complexity

The poor fit of the original factor structure proposed by Banner et al. (2023) suggests that the English SRIS-R structure cannot be directly transferred to the German adaptation without modifications. One possible explanation concerns linguistic and cultural differences affecting item interpretation. Several items proved difficult to translate into German while preserving their original nuance and level of abstraction. For example, the item “My behavior often puzzles me” has no direct German equivalent conveying the same degree of subtle self-observational ambiguity. Other items, such as “Often I find it difficult to make sense of the way I feel about things”, appear comparatively broad and unspecific in German, potentially increasing interpretational variability across respondents. Such translation-related differences may partially explain the structural misspecification observed in the confirmatory analyses. While Grant et al. (2002) defined *Self-Reflection* as “the inspection and evaluation of one’s thoughts, feelings, and behavior” and *Insight* as “the clarity of understanding of one’s thoughts, feelings, and behavior,” the German item formulations appear to shift these emphases slightly. Specifically, the items of the *Self-Reflection* subscale in German reflect a stronger focus on the willingness and tendency to reflect on one’s thoughts, feelings, and behavior, whereas the *Insight* subscale captures clarity of understanding and evaluation of these inner processes.

Although the bifactor model demonstrated comparatively good global fit indices, bifactor-specific diagnostics did not support the assumption of a strong overarching general factor. In particular, the explained common variance attributable to the general factor was relatively low (ECV = 0.38), and omega hierarchical for the general factor was modest (ωH = 0.29), indicating that only a limited proportion of reliable variance could be attributed to a common latent construct. In contrast, the group factors retained substantial reliable variance beyond the general factor, particularly the Insight factor. These findings suggest that the SRIS-R dimensions are not sufficiently explained by a dominant unitary construct despite the acceptable global fit of the bifactor model. This interpretation is also conceptually consistent with previous SRIS and SRIS-R research reporting only small to moderate associations between Self-Reflection and Insight (Banner et al., 2023; Grant et al., 2002; Silvia, 2021), supporting the notion that both dimensions represent related but distinguishable aspects of self-reflective functioning.

The exploratory factor analyses provided mixed evidence regarding the exact number of factors. Parallel analysis favored a three-factor solution, which also showed better overall fit than the two-factor EFA. However, the additional factor primarily reflected a subdivision of Self-Reflection into two substantially related components (*r* = 0.52), rather than a clearly distinct substantive dimension. We therefore retained the theoretically established distinction between Self-Reflection and Insight as the more parsimonious conceptual representation and focused subsequent model refinement on the two-factor structure. Importantly, the two-factor ESEM reproduced this broader distinction while allowing non-zero cross-loadings, suggesting that the superior fit of the three-factor exploratory solution may partly reflect item-level complexity and cross-loadings rather than necessarily indicating a substantively distinct third dimension. Nevertheless, the three-factor solution cannot be considered empirically disproven, and the present findings leave some uncertainty regarding the optimal item-level representation of the SRIS-R. Further research in independent samples is therefore needed to determine whether the observed complexity is better represented by additional substantive dimensions or by cross-loadings within the theoretically proposed two-factor structure.

Within this revised 13-item solution, Item 1 (“I am not really interested in analyzing my behavior”) was removed because it failed to demonstrate a salient loading on either factor. Conceptually, the item appears comparatively trivial, highly general, and potentially prone to socially desirable responding, particularly in psychologically informed samples. Its weak psychometric performance therefore suggests limited discriminative utility within the German adaptation. The revised parsimonious two-factor solution was subsequently retained as the practically preferred scoring model because it preserved the conceptual distinction between Self-Reflection and Insight while avoiding more complex specifications such as cross-loadings or correlated residuals. Although model fit indices remained below ideal thresholds, the structure appeared sufficiently interpretable and practically applicable for questionnaire use and scoring procedures.

To further examine the latent structure of the German SRIS-R, additional model refinements were explored. The best-fitting solution emerged when Item 5 (“I usually have a very clear idea about why I’ve behaved in a certain way”) was allowed to cross-load on both Self-Reflection and Insight. Conceptually, this finding appears plausible because the item simultaneously reflects active reflection on one’s behavior and perceived understanding of one’s own mental processes. Furthermore, the inclusion of a residual correlation between Items 11 (“I have a definite need to understand the way that my mind works”) and 14 (“It is important for me to be able to understand how my thoughts arise”) substantially improved model fit. Both items share highly similar wording and semantic content related to the motivation to understand internal cognitive processes, suggesting local item dependency beyond the common latent factor. Together, these findings indicate that the German SRIS-R may contain subtle overlaps between reflective engagement and insight-related content that are not fully captured by a strictly independent two-factor structure.

### 4.2 Practical scoring solution and conceptual continuity

Despite its comparatively weaker fit indices relative to the methodologically refined model, the parsimonious revised two-factor solution was considered the most suitable model for practical application and questionnaire scoring. In contrast to the methodologically refined solution, the parsimonious model does not require cross-loadings or correlated residuals and can therefore be implemented more easily in research and applied settings. This simplicity is particularly relevant for routine scoring procedures, comparability across studies, and future validation work. Conceptually, the parsimonious revised structure also appeared closer to the original SRIS-R framework. In the revised two-factor model, Self-Reflection and Insight remained positively but only weakly correlated, which is broadly consistent with previous SRIS and SRIS-R studies reporting small to moderate associations between both constructs (Banner et al., 2023; Grant et al., 2002; Silvia, 2021). In contrast, the methodologically refined model yielded a near-zero latent correlation between the factors, suggesting an almost complete statistical separation that may be less compatible with the original conceptualization of self-reflective functioning as partially interconnected processes.

The revised 13-item structure further demonstrated good internal consistency for both subscales, with internal consistencies comparable to those reported by Banner et al. (2023). Cronbach’s alpha coefficients for the revised German version were high for both Self-Reflection (*α* = 0.858) and Insight (*α* = 0.837), indicating satisfactory internal consistencies despite the shortened item set and structural modifications. Independent of the evidence regarding factorial structure, the revised subscale scores demonstrated satisfactory internal consistency. These reliability estimates concern score precision and should not be interpreted as evidence for superiority of the revised factorial structure.

Associations with external constructs provided differentiated evidence concerning the interpretation of the two subscale scores. HTMT analyses indicated a clearer differentiation between Self-Reflection and Insight in the revised structure compared with the original scoring structure (HTMT = 0.13 vs. 0.23). This pattern suggests that the revised German version may capture the distinction between both constructs more precisely.

The pattern of associations with external measures provided differentiated evidence regarding the nomological networks of Self-Reflection and Insight. Self-Reflection showed moderate positive associations with RRQ Reflection and motivation to gain true and accurate self-knowledge (SIM), consistent with its conceptualization as an active form of self-focused attention. At the same time, Self-Reflection also showed a small positive association with RRQ Rumination, resembling the pattern originally reported by Trapnell and Campbell (1999). Notably, the association between Self-Reflection and Rumination in the present study (*ρ* = 0.223) was comparable in magnitude to the association between reflection and rumination reported for the original RRQ. This pattern is theoretically plausible, as previous models conceptualize self-reflection as a self-focused feedback process that may involve both adaptive and maladaptive forms of self-attention (e.g., Grant, 2001; Grant et al., 2002). Prior research has similarly linked certain forms of self-focused cognition to rumination, neuroticism, and depressive symptoms (e.g., Ghorbani et al., 2003; Harrington and Loffredo, 2011). Thus, reflective engagement should not necessarily be interpreted as uniformly adaptive, but may become maladaptive when it shifts toward repetitive or evaluative rumination. Insight showed a distinct pattern, with moderate negative associations with RRQ Rumination and private self-consciousness, whereas associations with RRQ Reflection and SIM were close to zero. The negative association between Insight and Rumination is conceptually consistent with theoretical models distinguishing adaptive self-understanding from repetitive and maladaptive self-focused attention associated with psychological distress (Grant et al., 2002; Nakajima et al., 2017). Associations with the CFI Knowledge subscale were also close to zero across both scoring structures, replicating the weak associations with cognitive flexibility observed in the original English SRIS-R validation study (Banner et al., 2023). Overall, these findings suggest that Self-Reflection and Insight are embedded in partly distinct nomological networks. At the same time, several associations were weak or absent, and the observed pattern should therefore be interpreted as differentiated evidence regarding the external correlates of the two dimensions rather than as uniformly supporting convergent or discriminant validity.

### 4.3 Limitations and directions for future research

Although the study demonstrated initial evidence for theoretically expected associations with external constructs, these results must be interpreted in light of the specific measures and contexts applied. Consequently, it would be valuable for future studies to include additional validation methods, such as observer ratings or other external assessments, to complete self-report data and further examine the interpretation of SRIS-R scores. Moreover, studies assessing the test–retest reliability of the German SRIS-R are necessary to evaluate its stability and sensitivity to change over time. Longitudinal research is also recommended, both to provide additional evidence regarding the interpretation and applicability of SRIS-R scores and to explore the responsiveness of the SRIS-R to capture the development of self-reflection skills across clinical psychologists’ professional career and within targeted training programs aimed at enhancing these competencies.

Although the methodologically revised structure demonstrated substantially improved fit compared to the original model, RMSEA values remained slightly above conventional cutoff recommendations. Thus, the revised structure should not be interpreted as a definitive solution but rather as an empirically and theoretically improved approximation. Measurement invariance was not tested due to the homogeneity and imbalance of the sample. The vast majority of participants were female and enrolled in Bachelor’s psychology programs, resulting in insufficient and highly unequal subgroup sizes for reliable multi-group CFA, particularly with ordinal indicators and WLSMV estimation.

Although measurement invariance across gender and training stage could not be examined because of the pronounced subgroup imbalance in the derivation sample, the availability of the independent sample allowed us to test cross-sample measurement invariance. Scalar invariance was supported across the derivation and cross-validation samples. Nevertheless, future studies should specifically examine invariance across gender, training stage, clinical status, and German versus English language versions.

Lastly, future studies should seek to examine the measure’s applicability and generalizability in more heterogeneous samples, particularly with a broader age range and a more diverse professional background, ideally including practicing clinical psychologists and psychotherapists. Although self-reflection is considered a central professional competency in clinical psychology and psychotherapy, the present sample primarily consisted of psychology students at relatively early stages of training. Consequently, the findings may not fully generalize to licensed clinicians, whose self-reflective capacities and professional experiences may differ substantially from those of students. At the same time, this population remains highly relevant, particularly in the German context, where self-reflection constitutes an important component of university-based clinical psychology and psychotherapy degree programs. Therefore, assessing self-reflective processes already during academic and professional training represents a meaningful area of application for the German SRIS-R. To strengthen the findings of the present study, future research should additionally aim to increase sample sizes, as this would enhance statistical power and the robustness of the conclusions.

## 5 Conclusion

The present study provides the first psychometric examination of a German adaptation of the SRIS-R. The original factor structure could not be satisfactorily replicated, and exploratory analyses indicated greater item-level complexity than assumed by a strict simple-structure model. Nevertheless, ESEM supported the broader distinction between Self-Reflection and Insight, and the resulting 13-item structure showed a broadly comparable pattern of model fit, high factorial similarity, and scalar invariance in an independent sample. These findings provide initial cross-sample support for the two-dimensional interpretation while indicating that the precise item-level structure remains open to further investigation. Accordingly, the present findings do not support interpreting the SRIS-R primarily in terms of a total score. We therefore recommend using and interpreting the Self-Reflection and Insight subscale scores separately. Total-score reliability estimates are reported for comparability with previous studies but should not be taken as evidence that a unitary overall self-reflection score is psychometrically warranted.

Further validation across clinical populations, training stages, gender, and language versions is warranted.

## Data Availability

The raw data supporting the conclusions of this article will be made available by the authors, without undue reservation.

## Footnotes

1
https://osf.io/q8bha/overview?view_only=3d718f56588e4b0980ec792ffd964694
