# Supplementary material for: Psychometric properties of the self-reflection and insight scale (revised) in German-speaking clinical psychology trainees
Source: Front Psychol. 2026 Oct 16;17:1733344. doi: 10.3389/fpsyg.2026.1733344 (PMC13719603; doi:10.3389/fpsyg.2026.1733344)
Supplement: Supplementary file 1 [file Data_Sheet_1.PDF]

ESM 1. Questionnaire

Response form for the SRIS-R (German).

Selbstreflexions- und Introspektionsskala-Revised (SRIS-R)- deutsche Fassung

Modified version (13-item solution)

Subscales:

Self-reflection, items: 2, 4, 5, 6, 9, 11, 12, 14.

Insight, items: 3, 7, 8, 10, 13.

Original version (14-item solution)

Subscales:

Self-reflection, items: 1, 4, 6, 9, 11,12,14.

Insight, items: 2, 3, 5, 7, 8, 10, 13.

Geben Sie bitte für jede der folgenden Aussagen an, wie stark Sie ihr zustimmen oder nicht zustimmen. Versuchen Sie, genau und trotzdem zügig zu antworten.

[ ] stimme überhaupt nicht zu [ ] stimme nicht zu [ ] stimme eher nicht zu [ ] stimme eher zu [ ] stimme zu [ ] stimme voll und ganz zu

*1**	Ich bin *nicht* daran interessiert, mein Verhalten zu analysieren.
2	Ich bin ich mir über meine zugrundeliegenden Motive bewusst.
*3**	Ich bin häufig von der Art und Weise, wie ich Dinge empfinde, verwirrt.
4	Es ist mir wichtig, die Dinge, die ich tue, zu hinterfragen.
5	Normalerweise habe ich eine sehr klare Vorstellung davon, warum ich mich auf eine gewisse Art und Weise verhalten habe.
6	Ich bin sehr daran interessiert in den Blick zu nehmen, worüber ich nachdenke.
*7**	Wenn ich mir einem Gefühl bewusst werde, weiß ich oft nicht, welches es ist.
*8**	Manchmal weiß ich nicht, warum ich mich so verhalten habe wie, ich mich verhalten habe.
9	Mir ist es wichtig, meine Gefühle zu verstehen.
*10**	Wenn ich über meine zugrundeliegenden Motive nachdenke, verwirrt mich dies.
11	Ich habe das Bedürfnis zu verstehen, auf welche Art und Weise mein Verstand funktioniert.
12	Ich nehme mir häufig Zeit, um über meine Gedanken zu reflektieren.

*13**	Oftmals finde ich es schwierig, die Art und Weise wie ich fühle, zu verstehen.
14	Es ist mir wichtig zu verstehen, wie meine zugrundeliegende Motive entstehen.

Scoring*: Calculate a total score and subscale scores. Score 1 to 6, with 1= stimme überhaupt nicht zu, 2= stimme nicht zu, 3= stimme eher nicht zu, 4= stimme eher zu, 5= stimme zu, 6= stimme voll und ganz zu. Items indicated with * are to be reverse-coded (i.e. 6= stimme überhaupt nicht zu).*
