# Supplementary material for: Psychometric properties of the self-reflection and insight scale (revised) in German-speaking clinical psychology trainees
Source: Front Psychol. 2026 Oct 16;17:1733344. doi: 10.3389/fpsyg.2026.1733344 (PMC13719603; doi:10.3389/fpsyg.2026.1733344)
Supplement: Supplementary file 2 [file Data_Sheet_2.PDF]

ESM 2. Supplementary Tables

Supplementary Table 1
Descriptive item statistics for the original 14-item German SRIS-R.

Item	Mean (*SD*)	Skewness	Kurtosis
Item 1	4.51 (1.78)	-0.91	-0.75
Item 2	4.81 (0.85)	-1.41	3.68
Item 3	3.70 (1.24)	-0.42	-0.66
Item 4	4.67 (0.95)	-0.82	1.25
Item 5	4.48 (0.95)	-0.62	0.94
Item 6	4.83 (0.90)	-1.17	2.65
Item 7	3.97 (1.20)	-0.62	-0.22
Item 8	3.67 (1.16)	-0.33	-0.28
Item 9	5.34 (0.83)	-1.75	4.97
Item 10	4.19 (1.17)	-0.51	-0.27
Item 11	5.09 (0.90)	-1.19	2.51
Item 12	4.47 (1.18)	-0.60	-0.10
Item 13	3.84 (1.23)	-0.43	-0.44
Item 14	4.73	-1.01	1.54

Item	Mean<br>(*SD*)	Skewness	Kurtosis
	(1.04)		

Supplementary Table S2
Exploratory factor analysis loadings for the three-factor solution based on polychoric correlations.

Item	Factor 1	Factor 2	Factor 3
Item 1	.288	.213	−.111
Item 2	.060	−.052	.745
Item 3	.797	.015	−.109
Item 4	−.148	.290	.518
Item 5	.185	−.115	.715
Item 6	−.109	.324	.642
Item 7	.661	−.071	.049
Item 8	.586	−.131	.206
Item 9	.024	.716	.114
Item 10	.750	.103	.014
Item 11	.050	.893	−.055
Item 12	−.005	.286	.483
Item 13	.869	.039	.036
Item 14	−.033	.695	.098

Supplementary Table S3

Standardized factor loadings of the Parsimonious Revised and Methodologically Refined Two-Factor Models.

Factor	Item	λ Parsimonious	λ Refined
Self-Reflection	Item 2	.602	.608
	Item 4	.677	.701
	Item 5	.549	.499
	Item 6	.826	.851
	Item 9	.718	.741
	Item 11	.743	.660
	Item 12	.680	.692
	Item 14	.704	.620
Insight	Item 3	.749	.753
	Item 7	.675	.669
	Item 8	.633	.645
	Item 10	.771	.761
	Item 13	.876	.878
	Item 5 (crossloading)	—	.359

Note. λ = standardized factor loading. All primary factor loadings were statistically significant (*p*s < .001).
